# A New Microarchitecture-Based Parameter to Predict the Micromechanical Properties of Bone Allografts

**DOI:** 10.3390/ma16093349

**Published:** 2023-04-25

**Authors:** Zhuang Xiong, Léa Rouquier, Christine Chappard, Manon Bachy, Xingrong Huang, Esther Potier, Morad Bensidhoum, Thierry Hoc

**Affiliations:** 1 Université Paris Cité, CNRS, INSERM, ENVA, B3OA, 75010 Paris, France; 2 Department of Pediatric Orthopedic Surgery, Armand Trousseau Hospital, Assistance Publique–Hôpitaux de Paris, Sorbonne University, 75012 Paris, France; 3 Laboratory of Complex Systems, Ecole Centrale de Pékin, Beihang University, Beijing 100191, China; 4 Mechanical Department, MSGMGC, Ecole Centrale de Lyon, 69134 Ecully, France

**Keywords:** bone allografts, microarchitectural parameters, micromechanical parameters, finite element analysis

## Abstract

Scaffolds are an essential component of bone tissue engineering. They provide support and create a physiological environment for cells to proliferate and differentiate. Bone allografts extracted from human donors are promising scaffolds due to their mechanical and structural characteristics. Bone microarchitecture is well known to be an important determinant of macroscopic mechanical properties, but its role at the microscopic, i.e., the trabeculae level is still poorly understood. The present study investigated linear correlations between microarchitectural parameters obtained from X-ray computed tomography (micro-CT) images of bone allografts, such as bone volume fraction (BV/TV), degree of anisotropy (DA), or ellipsoid factor (EF), and micromechanical parameters derived from micro-finite element calculations, such as mean axial strain (ε_z_) and strain energy density (W_e_). DAEF, a new parameter based on a linear combination of the two microarchitectural parameters DA and EF, showed a strong linear correlation with the bone mechanical characteristics at the microscopic scale. Our results concluded that the spatial distribution and the plate-and-rod structure of trabecular bone are the main determinants of the mechanical properties of bone at the microscopic level. The DAEF parameter could, therefore, be used as a tool to predict the level of mechanical stimulation at the local scale, a key parameter to better understand and optimize the mechanism of osteogenesis in bone tissue engineering.

## 1. Introduction

Scaffolds are essential components of bone tissue engineering used either to repair large bone defects or to develop in vitro 3D human bone models [1,2]. Ideally, scaffolds for bone tissue engineering should have porosity, bone surface to volume ratio, mechanical behavior, and biocompatibility properties appropriate for the intended clinical application. Ideal scaffolds should also provide support for cell adhesion, and blood vessel and nerve growth [3]. Although autografts are considered the gold standard for bone grafts, their application is limited due to their restricted availability and associated morbidity [4]. Bone allografts and synthetic bone substitutes are the main alternatives to autografts. In clinical practice, bone allografts are widely used due to their accessibility and satisfactory osteoinductive and osteoconductive properties [5,6]. In addition to their role as space fillers, bone allografts are particularly used when the graft must also play a mechanical role in the reconstruction of bone loss.

The mechanical and structural characteristics of bone allografts closely resemble the in vivo bone tissue from which they are extracted. These characteristics can generally be described at the macroscopic scale (i.e., bone level) and at the microscopic scale (i.e., trabeculae level). While several studies have confirmed that the range of macroscopic strain required for the optimal functioning of bone tissue in vivo varies from 0.2% to 0.4% [7], little is known about the local solicitation experienced by the cells within allografts under physiological macroscopic strain. It is admitted that bone cells can perceive the mechanical stimulus of the local environment through the scaffold [8]. Furthermore, bone cells involved in the bone remodeling process are mechanosensitive cells whose behavior is influenced both by their biochemical environment and by the local mechanical stimulation [9,10,11,12]. It is therefore important to have a better understanding of the local mechanical environment of allografts as experienced by bone cells.

Many parameters originally developed to describe bone changes in osteoporosis can be used to characterize the microarchitecture of allografts, namely the bone volume fraction (BV/TV), the structure model index (SMI) [13], the degree of anisotropy (DA) [14], and more recently the ellipsoid factor (EF) [15]. BV/TV is the parameter most correlated with macroscopic mechanical properties, as it measures bone content [16]. DA measures the principal orientation of the substructure and quantifies its spatial distribution. SMI and EF are parameters used to estimate the distribution of plate-like and rod-like structures in the trabecular bone microarchitecture. However, the ability of SMI to describe the microarchitecture has been questioned due to its strong dependence on BV/TV and its potentially negative values [17].

The effects of different microarchitectural features of the scaffold on bone cell behavior have been investigated in the literature. It was found that the local mechanical conditions were modified due to local microarchitectural differences [18]. Similarly, cellular activity and bone-like tissue formation depended on the microarchitecture of the scaffold [19]. The local mechanical environment is generally described by the local strain and stress levels and strain energy density. The influence of microscopic mechanical stimulation on bone cell behavior has already been studied; in particular, the effects of strain level [20,21], stress level [22,23], and strain energy density [24].

The relationship between microarchitecture features and macroscopic mechanical properties of trabecular bone has been extensively studied [25,26,27]. However, to date and to the best of our knowledge, the relationship between the scaffold microarchitecture and the microscopic mechanical environment remains unknown. This knowledge, however, is central to determine the local mechanical stimulation that is actually applied to bone cells when a 3D, complex scaffold such as bone allograft is macroscopically loaded. At this microscale, previous studies [28,29] have illustrated that BV/TV, Tb.Sp, and Tb.N microarchitectural parameters were linked to local strain distribution. However, these parameters do not take into account the distribution of plates and rods or their orientation, which can be quantified through the DA, the SMI, and the EF parameters, and which can influence micromechanical properties. As mechanical stimulation can strongly affect cell behavior, filling this gap of knowledge will help better understand cell behavior. Hence, a predictive microarchitectural index of the micromechanical loading would be a major asset for scaffold development in bone tissue engineering.

The objective of the present study was, therefore, to investigate the relationship between microarchitectural parameters of bone allografts and the local mechanical properties. Conventional microarchitectural parameters were obtained from X-ray computed tomography (micro-CT) images and micromechanical parameters were derived from micro-finite element simulations. The hypothesis underlying this work is that BV/TV alone cannot fully predict micromechanical properties. A combination of microarchitectural parameters reflecting the distribution of plates and rods, as well as their orientation, is required to predict the local mechanical properties experienced by the cells.

## 2. Materials and Methods

### 2.1. Experimental Design

**Figure 1:**
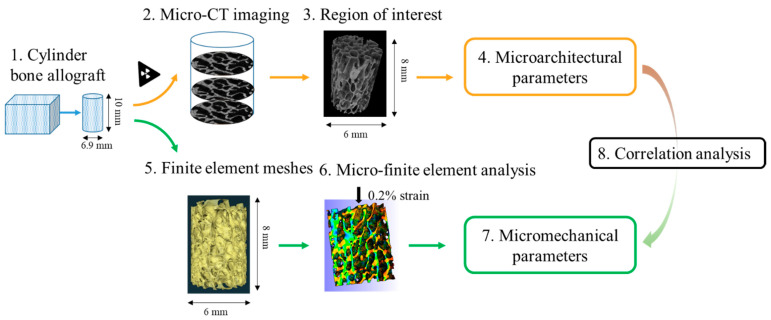
Methods overview: Step (1) Cylindrical samples (10 mm-height and 6.9 mm-diameter) were extracted from commercial cubic blocks obtained from a bone bank; Step (2) Cylindrical samples were scanned with micro-CT and 3D images were reconstructed; Step (3) A cylindrical region of interest (8 mm-height and 6 mm height) was selected for each sample, and the threshold of gray levels were calculated to binarize images; Step (4) For each sample, microarchitectural parameters were measured using CTAn software, except for EF, which was measured using Fiji software; Step (5) Micro-finite element meshes were generated for the region of interest for each sample using Avizo software; Step (6) Micro-finite element analysis was performed for each sample under 0.016 mm displacement, corresponding to a 0.2% uniaxial compressive strain, using FEBioStudio software; Step (7) For each sample, micromechanical parameters were calculated as the averaged values of all the micro-finite elements; Step (8) The relationship between the microarchitectural and micromechanical parameters obtained in Step 4 and Step 7, respectively, was studied.

To evaluate the correlation between microarchitecture features and the micromechanical properties within a scaffold requires a study coupling scaffold imaging and micro-finite element simulations. The scaffold analyzed in the present study is a bone allograft. This choice is related to several elements, including (i) bone allografts are classically used in the repair of bone defects in clinical practice; (ii) bone allografts come from bone banks and are therefore easily accessible for future studies; and (iii) bone allografts are harvested from human femoral heads and therefore correspond to a standard trabecular bone tissue microarchitecture. In addition, bone allografts are mineralized tissues that allow the use of X-ray micro-CT imaging at the micrometer scale. The high resolution of the micro-CT images enables both the determination of the scaffold microarchitectural parameters and the implementation of a micro-finite element simulation method. All these experimental and numerical methods applied on a large number of scaffolds (i.e., 29) provide a large amount of data that allows the implementation of a multilinear regression analysis and the emergence of key microarchitectural parameters in the prediction of the micromechanical loading. A detailed experimental design is illustrated in Figure 1.

### 2.2. Sample Preparation

Twenty-nine bone allografts were obtained from a bone bank (BioBank, 77127 Lieusaint, France) that collects trabecular bone from femoral heads of human donors. Bone tissue samples were devitalized and defatted using the Supercrit^®^ inactivation process and sterilized by gamma irradiation at 25 kGy [30]. Cylindrical allograft samples (6.9 mm diameter and 10 mm height) were cut from 20 × 10 × 10 mm trabecular bone blocks using a trephine in a water bath and then air-dried at room temperature.

### 2.3. X-ray Computed Tomography Analysis and Calculation of Bone Microarchitectural Parameters

Three dimensional microarchitectural parameters of each of the twenty-nine samples were obtained from micro-CT. Acquisitions were performed using the core beam Skyscan 1172 micro-CT (Bruker, Kontich, Belgium). The acquisition parameters were set to 40 kV, 100 µA, with a rotation angle of 0.3°, no filter addition, a voxel size of 7.9 × 7.9 × 7.9 µm with a frame averaging of 10. The scanned images were reconstructed with NRecon (v. 1.7.1.0, Bruker, Kontich, Belgium) and analyses were performed with CTAn (v. 1.17.7.2, Bruker, Kontich, Belgium) software. To avoid boundary effects, the measurements of microarchitectural parameters were performed on a central, cylindrical volume of 6 mm diameter and 8 mm height. The gray level threshold for bone tissue was obtained using the 3D auto-Otsu algorithm integrated in the CTAn software. In the present study, the following regular bone microarchitectural parameters were determined [31]: bone volume fraction (BV/TV; %), bone surface-to-volume ratio (BS/BV; mm^−1^), trabecular thickness (Tb.Th; mm), trabecular number (Tb.N; mm^−1^), trabecular separation (Tb.Sp; mm), structure model index (SMI), and degree of anisotropy (DA). A detailed description of micro-CT, as well as the definition of microarchitectural parameters, are given in the Supplementary Materials Section S1.

The microarchitecture of trabecular bone is usually compared to a structure composed of plates and rods, which can be characterized using SMI or the ellipsoid factor (EF). The CTAn software was used to calculate SMI, the value of which ranges from 0 to 3 to represent a predominant plate-like and rod-like microarchitecture, respectively. The BoneJ extension (BoneJ2) [32] in Fiji (v. 1.53) [33] free software was used to calculate EF, the value of which ranges from −1 to 1 to represent a predominant plate-like and rod-like microarchitecture, respectively. The same binarized images were used for both CTAn and BoneJ. The modified formula of DA was used in the present study with a range from 0 to 1, where 0 represents an isotropic structure.

The root mean square coefficient of variation [34], calculated from 3 different samples scanned 3 times, was used to evaluate the precision of the measurements for all microarchitectural parameters used in the present study.

### 2.4. Micro-Finite Element Meshing

**Figure 2:**
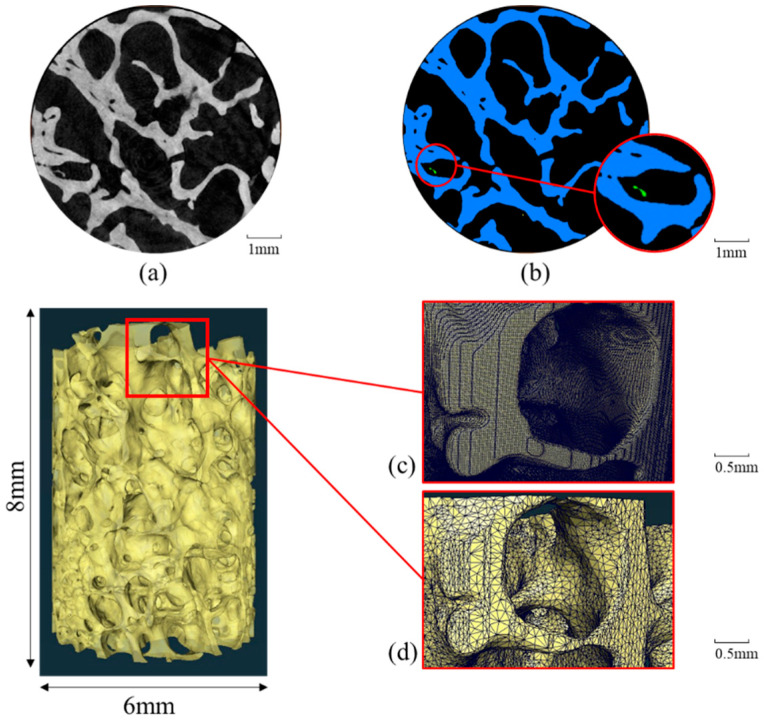
Finite element meshing process performed in Avizo^®^: (**a**) filtered image; (**b**) binarization and removal of unconnected component; (**c**) originally generated meshed surface; (**d**) simplified meshed surface.

The 3D micro-CT images were used to generate finite element meshes, as shown in Figure 2. The meshing process was performed using Avizo^®^ software (v. 2021.1, Berlin, Germany). The 3D images were converted to 8-bits and a 3D median filter of 3 voxels was applied to reduce noise and increase image contrast. Unconnected components were detected using Avizo^®^’s built-in label analysis method (Figure 2b) and were removed prior to surface generation. The original mesh surface (Figure 2c) was then simplified by the software, as shown in Figure 1d. A preliminary study was conducted specifically for this simplification step in order to maintain a good compromise between model accuracy and computational cost. In the end, the surface mesh was close to 2,000,000 facets and 500,000 nodes for each sample. A detailed description of the procedures for micro-finite element meshing is given in Supplementary Materials Section S2.

### 2.5. Micro-Finite Element Analysis

Tetrahedral meshes were generated and quasi-static uniaxial compression simulations along the z-axis of the cylindrical core were performed using FEBioStudio^®^ software (v. 1.9.0) [35]. All samples were subjected to a macroscopic compressive uniaxial strain level of 0.2%, corresponding to the physiological in vivo strain according to the literature [36,37]. Boundary conditions were defined such that all nodes on the upper surface of the bone core were displaced by 0.016 mm and those on the lower surface were fixed along the core axis. The bone phase was assumed to be an isotropic elastic material with a Young’s modulus E equal to 15 GPa and a Poisson’s ratio υ set at 0.3 [38,39,40]. A detailed description of micro-finite element analysis is given in Supplemental Materials Section S3. The finite element models used in the present study can be found in the Data Availability Statement section.

Different microscopic, i.e., trabeculae scale mechanical parameters were analyzed. The axial strain and stress in the direction of compression, denoted ε_z_ and σ_z_, respectively, were extracted from the results of micro-finite element simulations at 0.2% macroscopic strain. The von Mises stress σ_e_ and the strain energy density W_e_ were also calculated. A custom post-processing code written in Python 3.9 (Python Software Foundation, https://www.python.org/ (accessed on 10 October 2022)) was developed to calculate these parameters. For each sample, the average values of finite elements for each micromechanical parameter were calculated.

### 2.6. Statistical Analysis

For the Pearson’s correlation study, the representative sample size was defined assuming an expected correlation of 0.5, a two-tailed significance level (α) of 0.05, and a power level (1-β) of 80%. This calculation gave a sample size of twenty-nine (https://wnarifin.github.io/ssc_web.html (accessed on 15 March 2023)). Statistical analyses were performed using GraphPad Prism version 9.2.0 for Windows (GraphPad Software, San Diego, CA, USA, www.graphpad.com (accessed on 22 November 2022)). After checking for normal distribution using Shapiro-Wilk tests, Pearson regression coefficients were calculated to assess the correlation between the parameters. Multilinear regression analysis was performed to study the contribution of microarchitectural parameters in predicting the micromechanical parameters. In all cases, *p* values < 0.05 were considered significant.

## 3. Results

### 3.1. Trabecular Bone Microarchitectural Parameters

As two software were used to obtain the microarchitectural parameters, the BV/TV and DA values obtained from both CTAn and BoneJ were compared by a linear regression analysis. The BV/TV value of each sample measured by CTAn and BoneJ had a regression correlation coefficient R^2^ = 0.999; for DA, this value was equal to 0.978.

**Figure 3:**
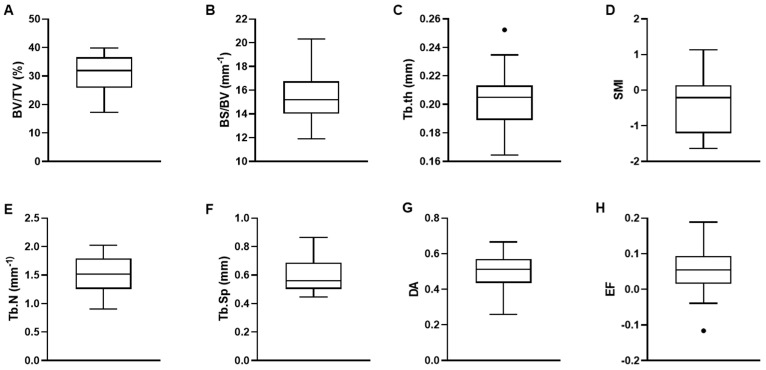
Box-whisker plots of microarchitectural parameters derived from CTAn, except for EF derived from Bone J: (**A**) Bone volume fraction (BV/TV); (**B**) Bone surface-to-volume ratio (BS/BV); (**C**) Trabecular thickness (Tb.Th); (**D**) Trabecular number (Tb.N); (**E**) Trabecular separation (Tb.Sp); (**F**) Structure model index (SMI); (**G**) Degree of anisotropy (DA); (**H**) Ellipsoid factor (EF). Box center lines, bounds of boxes, and whiskers indicate median, first and third quartiles, and minima and maxima within a 1.5 times interquartile range (IQR), respectively, points in the graph are outliers, N = 29.

Based on the correlation coefficient, the microarchitectural parameters obtained with CTAn and BoneJ software were considered similar, with the exception of EF, which could only be obtained in Bone J. These parameters for all the samples are shown in Figure 3. The measured mean values and standard deviations for the 29 samples were equal to 30.9 ± 6 for BV/TV; 15.5 mm^−1^ ± 1.8 for BS/BV; 0.2 mm ± 0.02 for Tb.Th; 1.5 mm^−1^ ± 0.3 for Tb.N; 0.6 mm ± 0.1 for Tb.Sp; −0.4 ± 0.77 for SMI; 0.5 ± 0.1 for DA; and 0.06 ± 0.06 for EF. It should be noted that the precision of the measurements was assessed by calculating the root mean square coefficient of variation (RMSCV; %) for all the microarchitectural parameters, which were less than 5%, except for EF and SMI, which had RMSCVs of 20% and 50%, respectively. All the microarchitectural parameters for individual samples are given in Supplementary Table S1.

Linear regression analyses were performed between all the microarchitectural parameters. The obtained regression coefficients R^2^ are shown in Table 1. Values greater than 0.5 are highlighted in gray. Tb.Th was linearly correlated (R^2^ = 0.81) with BS/BV. Tb.N was linearly correlated with BV/TV (R^2^ = 0.76). Tb.Sp was also linearly correlated with BV/TV (R^2^ = 0.76). In addition, SMI was linearly correlated with BV/TV (R^2^ = 0.83). In the analysis of the present study, DA and EF were found not to be linearly correlated—or only weakly linearly correlated—with the other parameters extracted from the microarchitecture.

### 3.2. Micromechanical Parameters

**Figure 4:**
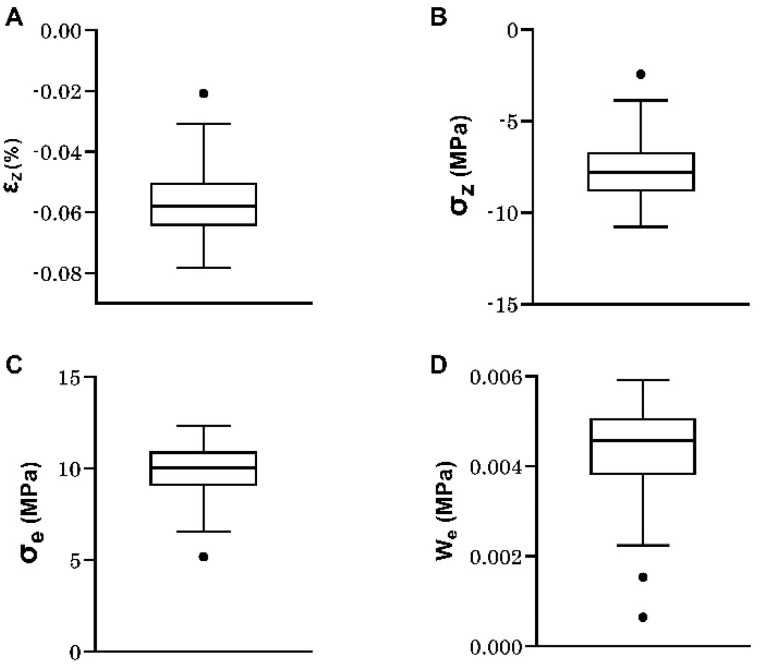
Box-whisker plots of micromechanical parameters derived from micro-finite element calculations: (**A**) Mean axial strain (ε_z_); (**B**) Mean axial stress (σ_z_); (**C**) Mean von Mises stress (σ_e_); (**D**) Mean strain energy density (W_e_). Box center lines, bounds of boxes and whiskers indicate median, first and third quartiles and minima and maxima within a 1.5 times interquartile range (IQR), respectively, points in the graph are outliers, N = 29.

For each of the 29 samples, numerical micro-finite element simulation allowed the characterization of the mean strain ε_z_ and mean stress σ_z_ in the compression axis, the mean von Mises stress σ_e_, and the mean strain energy density W_e_. All parameters were calculated by averaging all the elements of the meshes, and their values are shown in Figure 4. The mean values and standard deviations measured for all the samples were equal to −0.056% ± 0.013 for ε_z_, −7.7 MPa ± 1.95 for σ_z_, 9.9 MPa ± 1.66 for σ_e_, and 0.0043 MPa ± 0.0012 for W_e_. All the micromechanical parameters for individual samples are given in Supplementary Table S2.

### 3.3. Relationship between Microarchitectural and Micromechanical Parameters

Linear regression analyses were performed between the four micromechanical parameters derived from micro-finite element simulation and the different microarchitectural parameters measured with CTAn and BoneJ software. Table 2 shows the obtained linear regression coefficients. The results show that the microarchitectural parameters most linearly correlated with the mechanical parameters were EF and DA, two parameters related to the constituent distribution of the microarchitecture. However, only W_e_ was linearly correlated with EF with a coefficient greater than 0.5.

### 3.4. DAEF: An Index Derived to Predict Micromechanical Parameters

Since the two microarchitectural parameters most linearly correlated with the micromechanical parameters were EF and DA, it was assumed that an index derived from a linear combination of these two parameters could be highly linearly correlated with the micromechanical parameters. A multilinear regression analysis was performed for each of the four micromechanical parameters individually. Each micromechanical parameter Y was assumed to be described by the following linear equation:

(1) $$Y=\beta_{0}+\beta_{1}\cdot DA+\beta_{2}\cdot EF$$

where $\beta_{0}$, $\beta_{1}$, and $\beta_{2}$ are constants, and DA and EF the two microarchitectural parameters. These different parameters are given in Table 3 with the slope of the corresponding regression line $\beta_{2}$/$\beta_{1}$.

Since the ratios $\beta_{2}$/$\beta_{1}$ were in the same range of values for all the micromechanical parameters studied, an average value γ (equal to 1.69) was used to define the composite index of DA and EF, called DAEF:

(2) DAEF=DA+1.69·EF

**Figure 5:**
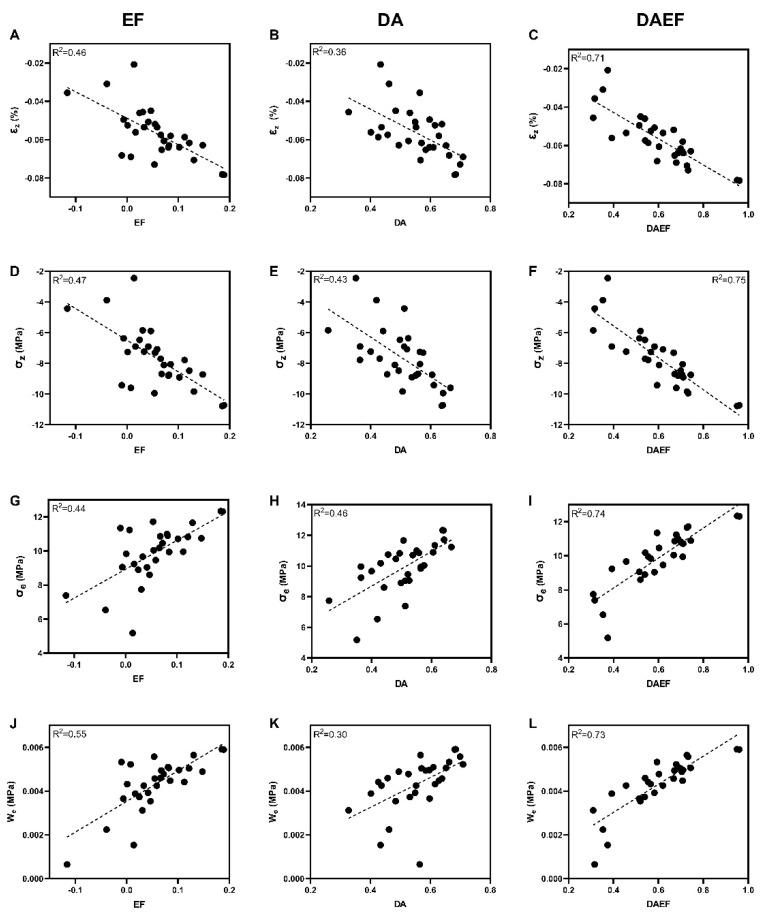
Linear regression plots between micromechanical parameters and ellipsoid factor (column 1), degree of anisotropy (column 2), and DAEF (column 3) for the 29 samples: (**A**) ε_z_-EF; (**B**) ε_z_-DA; (**C**) ε_z_-DAEF; (**D**) σ_z_-EF; (**E**) σ_z_-DA; (**F**) σ_z_-DAEF; (**G**)σ_e_ -EF; (**H**) σ_e_-DA; (**I**) σ_e_-DAEF; (**J**) W_e_-EF; (**K**) W_e_-DA; (**L**) W_e_- DAEF.

The DAEF index was calculated for all 29 samples. Figure 5 shows the linear correlation between each of the micromechanical parameters investigated in the present study, and DA, EF, and the new index DAEF.

## 4. Discussion

In the context of large bone defect repair or bone tissue engineering, micromechanical properties of the scaffold are understudied parameters. However, it is well established that the scaffold plays a key role in the transmission of mechanical loads from the macroscopic to the cellular level. In the present study, microarchitectural parameters, such as BV/TV, DA, and EF obtained from micro-CT images, and micromechanical parameters, such as strain, and stress derived from micro-finite element analysis, as well as their regression relationship, were investigated in bone allograft samples. While linear regression analyses showed weak correlations between these two types of parameters, a composite index called DAEF, derived from a linear combination of the DA and the EF, was found to significantly improve the analyzed linear regressions. This index, accessible by micro-CT imaging, allows the prediction of the local level of strain, stress, and strain energy density of the scaffold.

The values of the microarchitectural parameters of the samples were measured and compared with those reported in the literature. The results obtained in the present study are similar to those previously obtained in human trabecular bone [41,42]. Regarding the correlation between the microarchitectural parameters, the linear correlations depend on many factors, such as the sampling site and the age of the tissue [43]. It is therefore difficult to make direct comparison of the linear correlation obtained in the present study with the literature. For the samples used in the present study, it should be noted that the DA and the EF parameters were either not, or only weakly, correlated with other microarchitectural parameters, and there was no linear correlation between the DA and EF. This result shows that DA and EF are two independent parameters that describe different aspects of the microarchitecture of the scaffold.

To describe the distribution of plates and rods in the microarchitecture of trabecular bone, the SMI is widely used. Based on the variation in surface curvature, it has a theoretical value between 0 and 3 to represent a plate-like or rod-like structure, respectively. In the present study, and as reported in various other studies [17,44], the SMI was strongly negatively correlated with BV/TV. Therefore, it is difficult to use this parameter to dissociate the effect of microarchitecture organization from the effect of BV/TV. Furthermore, the measurement of the SMI is based on the assumption that the entire surface of the bone tissue is convex. The negative SMI values obtained in 60% of all the samples analyzed in the present study indicate that the bone surface of the samples is not purely convex. The negative values of SMI have been reported several times in the literature [45,46], which has led to great caution in the direct use of SMI to quantify the distribution of plates and rods. This is especially the case for femoral head sites which are not the primary site of osteoporotic fracture. It is therefore recommended to use EF as a parameter to describe the plate- or rod-like property of scaffolds, which was not influenced by BV/TV and had a better reliability than SMI in the present study.

The micromechanical parameters derived from finite element simulations are quite numerous, but are usually performed in order to predict the macroscopic behavior of bone tissue in the elastic domain [47,48,49], or to predict the damage or fracture features [50,51]. The mean strain, mean stress, and mean strain energy density of a scaffold at the local level have been so far poorly investigated under the macroscopic physiological strain level. Nevertheless, the calculation results of the present study can be compared with literature studies performed in the elastic domain. In particular, the averaged mean strain calculated in our study was four times smaller than the applied macroscopic strain, which is consistent with the literature [21,52]. As bone remodeling is thought to be mechanically governed by variations in the strain energy density, this parameter has been the subject of several studies at the local scale [24,53]. The values obtained in our study are in agreement with the others. This result suggests that a macroscopic strain of 0.2% allows a physiological level of local mechanical stimulation to be achieved.

Numerous studies have been conducted on the relationships between bone microarchitectural parameters and macroscopic mechanical properties, and are summarized in Table 4. In particular, several studies have demonstrated that BV/TV is a strong predictor of elastic modulus and/or yield strength at the macroscopic scale [13,14,19,39,44,45,47,51,54,55]. In contrast, studies investigating the relationship between microarchitectural and micromechanical parameters remain limited. Recently, with the development of 3D full-filed strain techniques, BV/TV, Tb.Sp, and Tb.N has been related to the local strain distribution for human humeral head or mouse tibia samples [28,29]. In the present study, the microarchitectural parameters DA and EF showed a stronger correlation with the micromechanical parameters than BV/TV, although the linear regression coefficient was rather low. According to the mathematical definition of these two parameters, the correlation result suggests that a more rod-like structure or a less isotropic structure leads to an overloaded mechanical environment. A linear combination of DA and EF, associating the shape of the trabeculae and the anisotropy of the microarchitecture, allowed for the definition of a new index, the DAEF. The high linear regression coefficient obtained with the micromechanical parameters and this index reported in our study shows a good predictive quality of the DAEF. Therefore, this index can be used as a method to quantify the influence of the microarchitecture of allografts on their micromechanical properties.

The present study has several limitations. The first limitation concerns the mechanical behavior of the material which was defined as homogeneous, linear, elastic, and isotropic. The heterogeneity of the local mechanical properties of the material was not taken into account in order to limit this study to the effect of different microarchitectures. Although it has been found in the literature that the model heterogeneity does not affect the macroscopic mechanical properties of the bone [56], the effect at the local scale remains unknown. A study will be performed in the future to quantify the effect of this heterogeneity on the predictive nature of the DAEF index, using an elastic modulus whose value varies according to the gray level variation observed within the scaffold structure using micro-CT. The second limitation of the present study lies in the parameters used to describe the local mechanical environment. The average values over the entire scaffold derived from micro-finite element simulation were used. Although these parameters are able to reflect the overall level of mechanical stimulation in the microarchitecture at the local level, the stimulation information at the single cell level was not reflected. Furthermore, at the cellular level, the characteristics of the mechanical stimulation—in particular, the areas of tension and compression—should be considered differently, as they lead to different cellular behaviors. Finally, the samples used in this study were allografts from a bone bank without specific orientation. They were taken from human femoral heads and processed to remove living tissue. Although the predictive character of the DAEF index is currently limited to this type of specimen, it could easily be adapted to bone tissue from other pathological or non-pathological harvesting sites or to other biomaterials.

In conclusion, the present study proposes a new DAEF index that combines the two microarchitecture parameters DA and EF to predict the micromechanical properties of trabecular bone. Hence, for the first time, the DAEF index obtained in the present study provides a predictive tool of micromechanical properties of bone allografts and allows determination of the influence of the microarchitecture on the loading experienced by the cells. The DAEF is strongly correlated with the micromechanical parameters calculated using micro-finite element simulations. It may therefore be a useful tool for optimizing scaffolds for the repair of large bone defects, or for developing in vitro 3D bone models where local mechanical properties are key elements. To further validate the relationship found in the current study, a heterogeneous material model will be developed in the near future to account for the tissue mechanical properties of individual trabeculae. In addition, the same procedures could be used to study the influence of different pathological microarchitectures on the local mechanical properties, such as osteoporotic bone, which remains a major challenge.

## Figures and Tables

**Table 1:** Linear regression analysis performed between the different parameters derived from microarchitecture. The regression coefficients R2 of each linear regression are reported. The values greater than 0.5 are highlighted in gray.

R^2^	BV/TV (%)	BS/BV (mm^−1^)	Tb.Th (mm)	Tb.N (mm^−1^)	Tb.Sp (mm)	SMI	DA	EF
BV/TV (%)	1							
BS/BV (mm^−1^)	0.26	1						
Tb.Th (mm)	0.08	0.81	1					
Tb.N (mm^−1^)	0.76	<0.01	0.05	1				
Tb.Sp (mm)	0.76	0.01	<0.01	0.88	1			
SMI	0.83	0.32	0.06	0.63	0.47	1		
DA	<0.01	0.01	0.03	0.01	<0.01	0.01	1	
EF	0.45	0.14	0.11	0.26	0.37	0.33	0.04	1

**Table 2:** R^2^ values obtained between microarchitectural parameters and micromechanical parameters. Microarchitectural parameters are ranked from most linearly correlated to least correlated. The values greater than 0.5 are highlighted in gray.

R^2^	ε_z_ (%)	σ_z_ (MPa)	σ_e_ (MPa)	W_e_ (MPa)
EF	0.46	0.48	0.44	0.55
DA	0.36	0.40	0.42	0.30
BV/TV (%)	0.29	0.28	0.26	0.26
SMI	0.32	0.22	0.23	0.28
Tb.Sp (mm)	0.26	0.26	0.27	0.25
Tb.N (mm^−1^)	0.16	0.14	0.14	0.13
BS/BV (mm^−1^)	0.14	0.14	0.10	0.12
Tb.Th (mm)	0.11	0.12	0.09	0.11

**Table 3:** Multilinear regression coefficients obtained for different micromechanical parameters.

	$\beta_{0}$	$\beta_{1}$	$\beta_{2}$	$\beta_{2}/\beta_{1}$
ε_z_	−0.016	−0.067	−0.117	1.74
σ_z_	−1.3	−10.6	−17.3	1.63
σ_e_	4.3	9.4	14.0	1.49
W_e_	0.0009	0.0054	0.0120	2.29

**Table 4:** Recent advances in studying the relationship between microarchitectural parameters and mechanical parameters (M = male, F = female, OP = osteoporotic, OA = osteoarthritis).

Refs.	Species	Sampling Site	Sample Number	Sex	Age	Pathology	Microarchitectural Parameters	Mechanical Parameters
Macroscopic scale								
[13]	Human	Femoral heads	77	29 M, 48 F	55–87 years old	OP	BV/TV, BS/BV, Tb.Th, Tb.Sp, Tb.N, SMI, DA	Maximum stress, elasticity stress, modules of elasticity, microcrack surface density
			25	10 M, 15 F	56–78 years old	OA	BV/TV, BS/BV, Tb.Th, Tb.Sp, Tb.N, SMI, DA	Maximum stress, elasticity stress, modules of elasticity, microcrack surface density
[14]	Human	Lumbar spines (L1–L5)	21	11 M, 10 F	65–86 years old	11 normal, 10 OA	BV/TV, Tb.N, SMI	Initial failure load, initial stiffness, post-fracture load, post-fracture stiffness
[19]	Human	Spine, femur	41	8 M, 7 F,	36–83 years old	Metastasis cancer	BV/TV, BS/TV, BS/BV, SMI, Tb.N, Tb.Th, DA	Modulus of elasticity, yield strain, yield strength
			96	22 M, 21 F	23–93 years old	Noncancer	BV/TV, BS/TV, BS/BV, SMI, Tb.N, Tb.Th, DA	Modulus of elasticity, yield strain, yield strength
[39]	Human	Femoral heads	42	7 M, 35 F	68–92 years old	OP	BV/TV	Modulus of elasticity, yield stress
[44]	Human	L2 vertebrae	23	8 M, 15 F	54–93 years old	/	BV/TV, Tb.Th, Tb.N, Tb.Sp, SMI, DA	Microcrack density, mean crack length, diffuse damage area
[45]	Rat	Left tibiae	10	F	18 weeks old	Sham operated	BV/TV, Tb.Th, Tb.Sp, Tb.N, SMI, DA	Modulus of elasticity, Shear modulus
			40	F	18 weeks old	Ovariectomy	BV/TV, Tb.Th, Tb.Sp, Tb.N, SMI, DA	Modulus of elasticity, Shear modulus
[47]	Human	Femoral heads	32	F	62–88 years old	/	BV/TV	Modulus of elasticity, stiffness
			26	F	73–87 years old	OP	BV/TV	Modulus of elasticity, stiffness
[51]	Porcine	Vertebrae	30	/	12–18 months old	/	BV/TV, BS/BV, Tb.Th, DA	Elastic stiffness, stress amplitude, fatigue life, accumulated damage
[54]	Human	Femur epiphyses	4	/	42–79 years old	Normal	BV/TV, Tb.N, Tb.Th, Tb.Sp, SMI, DA	Modulus of elasticity, ultimate stress, mechanical anisotropy
		Femoral heads	7	/	42–79 years old	OP	BV/TV, Tb.N, Tb.Th, Tb.Sp, SMI, DA	Modulus of elasticity, ultimate stress, mechanical anisotropy
		Femoral heads	12	/	42–79 years old	QA	BV/TV, Tb.N, Tb.Th, Tb.Sp, SMI, DA	Modulus of elasticity, ultimate stress, mechanical anisotropy
[55]	Human	Femoral heads and necks	45	M	52–71 years old	OA, Type 1 diabetes	BV/TV	Modulus of elasticity, yield stress, ultimate stress, post-yield toughness, toughness
Microscopic scale								
[28]	Human	Humeral heads	6	3 M, 3 F	54–82 years old	OA	BV/TV, Tb.N, Tb.Sp	Third principal strain
[29]	Mouse	Left tibia	7	F	16 weeks	/	BV/TV, Tb.Th, Tb.Sp, Tb.N	Maximal principal strain, minimal principal strain

## Data Availability

The 29 micro-finite element models of bone allografts used in the present study can be found at the following site in a FEBio format: https://github.com/gabixz/bone-allografts-febio-models (accessed on 20 February 2023). A Readme file is available for the description of the models.

## Supplementary Materials

The following supporting information can be downloaded at https://www.mdpi.com/article/10.3390/ma16093349/s1: Sections S1–S3: detailed description of the methods; Figure S1: reconstructed three-dimensional model of a sample; Figure S2: micro-finite element analysis of a sample; Table S1: microarchitectural parameters for 29 samples obtained from micro-CT images; Table S2: micromechanical parameters for 29 samples obtained from micro-finite element analysis.
