# Supplementary material for: A New Microarchitecture-Based Parameter to Predict the Micromechanical Properties of Bone Allografts
Source: Materials (Basel). 2023 Apr 25;16(9):3349. doi: 10.3390/ma16093349 (PMC10179528; doi:10.3390/ma16093349)
Supplement: Supplementary file 1 [file materials-16-03349-s001.zip › materials-2266319-supplementary.pdf]

SUPPLEMENTARY MATERIALS

S.1 X-ray computed tomography analysis and calculation of bone microarchitectural parameters

X-ray computed tomography analysis (micro-CT) is an X-ray attenuation method that provides three-dimensional, high-resolution, non-destructive analysis of the density, microarchitecture and morphometry of hard, mineralized tissues such as bone and teeth. During the scan, radiograph images are taken of the sample at multiple angles. The acquisition parameters used during the scanning process were set to 40 kV, 100μA, the rotation angle of 0.3°, the exposure time of 375 ms, the frame averaging of 10, the random movement of 15, a voxel size of 7.9*7.9*7.9 μm, and with no filter addition. The radiograph images were then processed using a back-projection algorithm that combines all the information to give a stack of cross-sectional 8-bits gray level images of each sample with integrated software NRecon (v. 1.7.1.0). These reconstructed images are then processed using a 3D rendering software CTAn (v. 1.17.7.2) that creates 3D models for the measurements of bone morphometry and density. Prior to the measurement, a cylindrical region of interest with a diameter of 6 mm and a height of 8 mm was selected for the stack of reconstructed images of each sample to avoid boundary effects. To measure the microarchitectural parameters, the 8-bits gray-level images were binarized with the thresholds calculated by CTAn integrated 3D auto-Otsu algorithm, where white pixels represented for bone tissue and black pixels represented for the exterior space.

In the present study, microarchitectural parameters were measured with software CTAn (v. 1.17.7.2) except for ellipsoid factor (EF), which was measured with BoneJ extension (BoneJ2) [29] in Fiji (v. 1.53). The same stack of binarized images were used for each sample during the measurement of the parameters.

All the microarchitectural parameters are defined as follows [29]:

BV/TV (%): the ratio between bone volume (BV) and tissue volume (TV), BV was calculated using tetrahedrons corresponding to the enclosed volume of the triangulated surface, TV is the total volume examined per sample;

BS/BV (mm^{-1}): the ratio between bone surface (BS) and bone volume (BV), BS was calculated using the marching cubes method to triangulate the surface of the mineralized phase;

Tb.Th (mm): a model-independent three-dimensional thickness, a local value for a point in solid is defined as the diameter of the largest sphere which is entirely bounded within the solid surfaces and encloses the point;

Tb.N (mm^{-1}): the inverse of the mean distance between the middle axes of the structure;

Tb.Sp (mm): a model-independent three-dimension thickness same as Tb.Th, but measures the distances of spaces;

SMI: calculated from BV and BS before and after one pixel dilation (BS_{dil}) as in the following equation:

$$SMI=6*[(BS_{dil}*BV)/BS^2] \quad (S1)$$

DA: defines the orientation of the structure evaluated by fitting the mean intercept length (MIL) points to an ellipsoid, it is define as in the following equation:

$$DA = 1-MIL3/MIL1 \quad (S2)$$

where MIL1 is the largest MIL and MIL3 is the smallest MIL.

EF: measures the plate-and-rod distribution of the structure by fitting ellipsoids into the 3D structure [18].

S.2 Micro-finite element meshing

The 3D micro-CT images of the cylindrical region of interest with a diameter of 6 mm and a height of 8 mm for each sample were next used to generate micro-finite element meshes with Avizo® software (v. 2021.1). The images were treated with the built-in modules prior to mesh generation. Firstly 3D "median filter" of 3 voxels was applied to each image stack to reduce the noises in the images. Then an "interactive thresholding" module was applied for the gray-level images to binarize the images with the gray-level threshold obtained beforehand with 3D auto-Ostu algorithm.

Next a “binary smoothing” module was used to smooth the boundary of binarized images. A 3D “label analysis” module was followed to detect all the separate volumes existed in the total 3D volume. Afterwards a “remove small spots” module was used to remove all the volumes which is not connected to the main structure. Then the “remove island” and “smooth label” modules were applied to the image stack in the Segmentation surface. Finally, the “generate surface” module was used to generate micro-finite element surface meshes for each sample. By default, in Avizo® the original generated meshes are of great density, which is not directly applicable for micro-finite element analysis. Avizo® provides a built-in “simplification” option for the generated meshes. A preliminary study was conducted specifically for this simplification step in order to maintain a good compromise between model accuracy and computational cost. In the end, the surface mesh was close to 2,000,000 facets and 500,000 nodes for each sample.

S.3 Micro-finite element analysis

The generated surface meshes for each sample in Avizo® were used to generate tetrahedral meshes with FEBioStudio® software (v. 1.9.0). Quasi-static uniaxial compression simulations along the z-axis of the cylindrical core were performed and all the cylindrical samples were subjected to a macroscopic compressive uniaxial strain level of 0.2%, corresponding to the physiological in vivo strain. The material for bone phase was assigned as “isotropic elastic” which already existed in FEBio (v. 3.8), with a Young’s modulus E equal to 15 GPa and a Poisson’s ratio υ set at 0.3. For the boundary conditions applied to the micro-finite element model, all the nodes on the lower surface of the cylinder were set to be fixed in the z-axis direction while all the nodes on the upper surface of the cylinder were set to have a displacement of 0.016 mm along the negative z-axis direction to simulate the 0.2% macroscopic strain. The 0.016 mm corresponds to the 0.2% of the height of the cylindrical micro-finite element model. A built-in linear solver in FEBio (v. 3.8) was used to perform the micro-finite element analysis.

During the micro-finite element analysis, the axial strain and stress in the direction of compression, denoted ε_z and σ_z, the von Mises stress σ_e, and the strain energy density W_e were calculated for every finite element. For each sample, the average value of these micromechanical parameters for all the finite elements were calculated to represent the mechanical stimulation experienced at the trabeculae scale. A custom post-processing code written in Python 3.9 (Python Software Foundation, https://www.python.org/) was developed to calculate these parameters.

A reconstructed 3D model of a sample is illustrated in Figure S1. The micro-finite element analysis of a sample is shown in Figure S2.

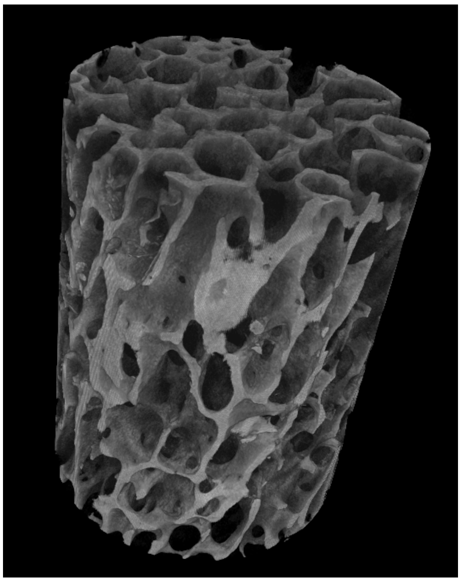

Supplemental Figure S1. Reconstructed three-dimensional image of an allograft sample. The finite element model corresponds to the cylindrical region of interest with a diameter of 6 mm and a height of 8 mm.

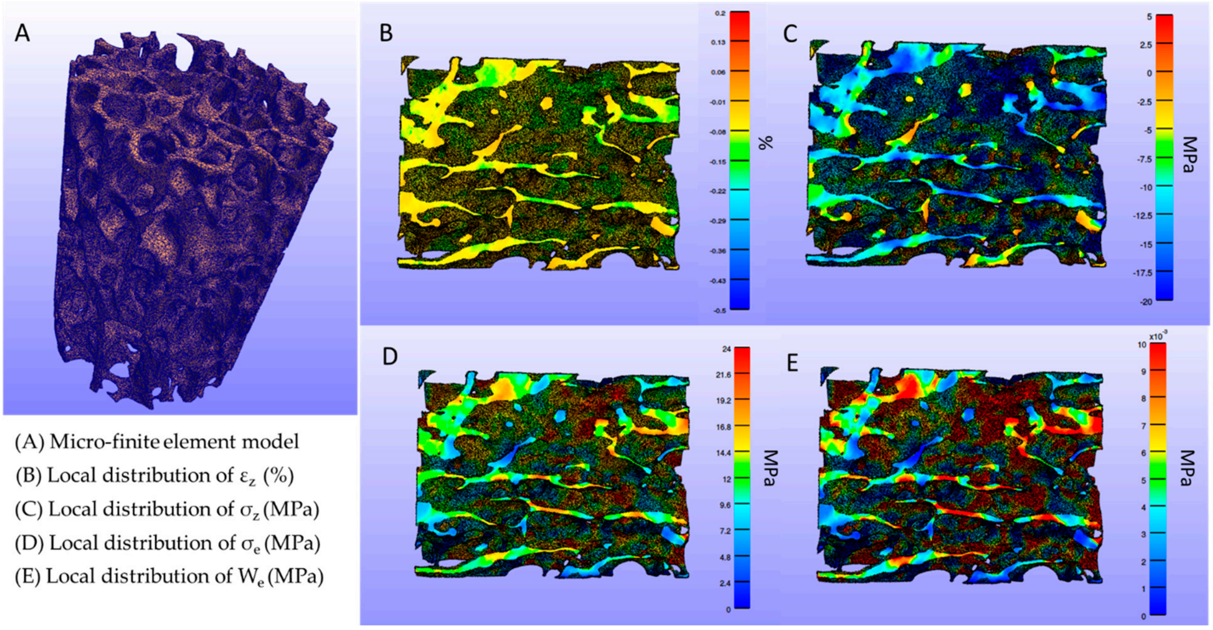

Supplemental Figure S2. Local distribution of micromechanical parameters of a bone allograft sample: A) micro-finite element model of the sample, B) the local axial strain (ε_z) distribution of the sample, C) the local axial stress (σ_z) distribution of the sample, D) the local von Mises stress (σ_e) distribution of the sample, E) the local strain energy density (W_e) distribution of the sample.

The obtained microarchitectural parameters from micro-CT images for all the 29 bone allografts are listed in Table S1. The calculated micromechanical parameters obtained from finite-element analysis are listed in Table S2.

Supplemental Table S1. Microarchitectural parameters measured for each bone allograft samples. For each sample: BV/TV, BS/BV, Tb.Th, Tb.N, Tb.Sp, SMI, and DA were calculated with CTAn software (v. 1.17.7.2); EF was calculated with BoneJ2 extension in Fiji (v. 1.53); DAEF = DA + 1.69 * EF.

N° sample	BV/TV (%)	BS/BV (mm^{-1})	Tb.Th (mm)	Tb.N (mm^{-1})	Tb.Sp (mm)	SMI	DA	EF	DAEF
**1**	24.86	15.44	0.21	1.19	0.69	0.22	0.67	0.008	0.68
**2**	24.92	16.44	0.20	1.26	0.68	0.33	0.61	-0.010	0.59
**3**	28.41	14.12	0.23	1.21	0.64	0.13	0.56	0.0849	0.71
**4**	29.45	14.39	0.23	1.29	0.62	0.05	0.54	0.102	0.71
**5**	39.08	13.93	0.21	1.90	0.47	-1.46	0.51	0.042	0.58
**6**	38.28	15.20	0.19	2.03	0.48	-1.64	0.26	0.031	0.31
**7**	35.89	14.57	0.21	1.72	0.51	-1.11	0.46	0.148	0.70
**8**	24.71	14.88	0.21	1.20	0.79	-0.01	0.50	0.025	0.54
**9**	27.86	15.67	0.20	1.41	0.64	-0.17	0.56	0.001	0.57
**10**	32.77	17.45	0.18	1.86	0.49	-0.62	0.56	0.068	0.67
**11**	33.17	15.36	0.20	1.65	0.55	-0.75	0.61	0.082	0.74
**12**	32.23	15.80	0.20	1.60	0.51	0.06	0.58	0.055	0.67
**13**	30.97	16.66	0.19	1.65	0.54	-0.29	0.40	0.033	0.46
**14**	32.44	13.50	0.23	1.39	0.64	-0.19	0.48	0.072	0.60
**15**	33.00	16.84	0.19	1.75	0.53	-0.85	0.55	0.081	0.69

**16**	32.06	18.90	0.17	1.86	0.48	-0.21	0.43	0.066	0.54
**17**	37.93	15.10	0.21	1.80	0.45	-0.64	0.49	0.121	0.70
**18**	24.99	20.33	0.16	1.52	0.56	0.61	0.44	0.047	0.52
**19**	27.29	14.60	0.22	1.25	0.68	0.03	0.64	0.053	0.73
**20**	21.61	17.06	0.19	1.12	0.77	0.37	0.52	0.059	0.62
**21**	39.80	13.82	0.21	1.88	0.48	-1.53	0.64	0.185	0.95
**22**	37.36	14.10	0.21	1.78	0.52	-1.31	0.36	0.112	0.55
**23**	26.64	16.26	0.19	1.41	0.69	-0.29	0.53	-0.006	0.51
**24**	31.90	13.78	0.21	1.49	0.69	-1.46	0.36	0.017	0.39
**25**	17.20	17.49	0.19	0.91	0.87	1.14	0.35	0.014	0.37
**26**	24.13	17.43	0.18	1.33	0.71	0.53	0.51	-0.116	0.32
**27**	27.53	13.94	0.23	1.22	0.72	0.15	0.42	-0.039	0.35
**28**	39.07	11.90	0.25	1.55	0.54	-1.44	0.51	0.130	0.73
**29**	39.64	13.95	0.21	1.93	0.45	-1.58	0.64	0.189	0.96

Supplemental Table S2. Micromechanical parameters calculated for each bone allograft samples. Each micromechanical parameter value corresponds to the average value of all the finite elements.

**N° sample**	ε_z (%)	σ_z (MPa)	σ_e (MPa)	W_e (MPa)
**1**	-0.069	-9.60	11.24	0.0052
**2**	-0.068	-9.42	11.35	0.0053
**3**	-0.058	-8.06	9.94	0.0045
**4**	-0.064	-8.91	10.71	0.0050
**5**	-0.051	-6.91	9.04	0.0039
**6**	-0.046	-5.85	7.74	0.0031
**7**	-0.063	-8.72	10.75	0.0049
**8**	-0.046	-6.47	8.90	0.0037
**9**	-0.052	-7.26	9.84	0.0043
**10**	-0.065	-8.69	10.86	0.0049
**11**	-0.063	-8.75	10.90	0.0051
**12**	-0.052	-7.31	10.04	0.0046
**13**	-0.054	-7.23	9.67	0.0042
**14**	-0.061	-8.11	10.46	0.0048
**15**	-0.064	-8.80	11.00	0.0051
**16**	-0.057	-7.70	10.18	0.0046
**17**	-0.062	-8.48	10.83	0.0050
**18**	-0.045	-5.90	8.60	0.0035
**19**	-0.073	-9.95	11.72	0.0056
**20**	-0.054	-7.09	9.46	0.0042
**21**	-0.078	-10.77	12.34	0.0059
**22**	-0.059	-7.79	9.96	0.0044
**23**	-0.050	-6.37	9.05	0.0037
**24**	-0.056	-6.90	9.24	0.0039
**25**	-0.021	-2.45	5.18	0.0015
**26**	-0.036	-4.43	7.39	0.0006
**27**	-0.031	-3.88	6.54	0.0022
**28**	-0.071	-9.84	11.66	0.0056
**29**	-0.078	-10.73	12.31	0.0059
